# Assessment of acetylcholinesterase activity in CD9-positive exosomes from patients with Parkinson’s disease

**DOI:** 10.3389/fnagi.2024.1332455

**Published:** 2024-02-07

**Authors:** Sumin Jeong, Kyu Hwan Shim, Danyeong Kim, Heewon Bae, Da-Eun Jeong, Min Ju Kang, Seong Soo A. An

**Affiliations:** ^1^Department of Bionano Technology, Gachon University, Seongnam, Republic of Korea; ^2^Department of Neurology, Veterans Health Service Medical Center, Veterans Medical Research Institute, Seoul, Republic of Korea

**Keywords:** Parkinson’s disease, blood, acetylcholinesterase, exosomes, CD9, cholinergic dysfunction

## Abstract

**Introduction:**

Parkinson’s disease (PD) is a neurodegenerative disorder characterized by dopaminergic dysfunction and associated with abnormalities in the cholinergic system. However, the relationship between PD and cholinergic dysfunction, particularly in exosomes, is not fully understood.

**Methods:**

We enrolled 37 patients with PD and 44 healthy controls (HC) to investigate acetylcholinesterase (AChE) activity in CD9-positive and L1CAM-positive exosomes. Exosomes were isolated from plasma using antibody-coupled magnetic beads, and their sizes and concentrations were assessed using transmission electron microscopy, nanoparticle tracking analysis, and western blotting. Subsequently, the AChE activity in these exosomes was analyzed in relation to various clinical parameters.

**Results:**

A significant decrease in AChE activity was observed in CD9-positive exosomes derived from patients with PD, whereas no significant differences were found in L1CAM-positive exosomes. Further analysis with a larger sample size confirmed a substantial reduction in AChE activity in CD9-positive exosomes from the PD plasma, with moderate diagnostic accuracy. The decrease in AChE activity of CD9-positive exosomes did not show an association with cognitive impairment but displayed a trend toward correlation with PD progression.

**Discussion:**

The reduction in AChE activity in CD9-positive exosomes suggests potential peripheral cholinergic dysfunction in PD, independent of the central cholinergic system. The observed alterations in AChE activity provide valuable insights into the association between cholinergic dysfunction and the pathogenesis of PD.

## 1 Introduction

Parkinson’s disease (PD) is characterized by the loss of dopaminergic neurons in the substantia nigra region and the presence of abnormal α-synuclein aggregates known as Lewy bodies, which are recognized as pathological hallmarks in the brains of patients with PD (Gibb and Lees, 1988; Spillantini et al., 1997). Efforts have been made to identify biomarkers for PD by measuring the concentration of specific α-synuclein variants in bodily fluids or peripheral tissues (Fayyad et al., 2019). However, it remains difficult to determine whether these biomarkers accurately reflect the brain pathology associated with PD (Ganguly et al., 2021).

Exosomes are a type of extracellular vesicle with diameters ranging from 50 to 150 nm that are released from the cell membrane upon fusion with late endosomes or multivesicular bodies (Doyle and Wang, 2019). Exosomes play a crucial role in cell-to-cell communication by facilitating bidirectional transfer of genetic and protein components, thereby influencing cellular functions (Zhang et al., 2019). Studies focusing on PD have provided evidence that pathogenic α-synuclein forms can spread through exosomes, potentially contributing to the progression of brain pathology (Tofaris, 2017). Exosomes derived from cerebrospinal fluid of individuals with PD and dementia with Lewy bodies demonstrated a higher abundance of α-synuclein species and the ability to induce α-synuclein oligomerization in recipient cells (Stuendl et al., 2015). Moreover, direct inoculation of exosomes derived from brains with Lewy body pathology into the hippocampus of normal mice triggered the aggregation of endogenous α-synuclein (Ngolab et al., 2017). The exploration of biomarkers for PD included the investigation of body fluid-based markers such as α-synuclein oligomers and total tau measurements (Ganguly et al., 2021). Several studies have reported elevated levels of α-synuclein in L1CAM positive exosomes derived from the blood of patients with PD compared to healthy controls (HC) (Lemprière, 2020). These findings highlight the potential of exosomes as pathophysiological and disease biomarkers of PD.

Acetylcholinesterase (AChE) is an enzyme primarily produced in postsynaptic neuromuscular junctions, especially in muscles and nerves (Trang and Khandhar, 2022). AChE activity serves as an indicator of cholinergic function and as a marker for evaluating neuronal functioning (Fedorova et al., 2015). A previous study showed that exosomal AChE activity was reduced in the blood of patients with PD compared to that in HC, with a negative correlation between exosomal AChE activity and disease progression (Shim et al., 2021). Cholinergic dysfunction has been implicated in the central and peripheral nervous systems of patients (Fedorova et al., 2015; Bohnen et al., 2018). Cholinergic imbalances, which are characterized by decreased cholinergic activity, have been observed in specific brain regions and peripheral tissues of individuals with PD. Cholinergic PET studies have demonstrated cholinergic denervation in the medial secondary occipital cortex of patients with PD and dementia (PDD) (Müller and Bohnen, 2013). Additionally, 11C-donepezil PET has confirmed cholinergic loss and provided evidence of parasympathetic denervation in the peripheral nervous system of patients with PD (Gjerløff et al., 2014; Tatyana et al., 2017). The etiology of cholinergic dysfunction in PD is not fully understood; however, exosome studies may offer insights into the pathophysiology of cholinergic system imbalance in PD.

In this study, exosomes were isolated using magnetic beads conjugated with antibodies, enabling more specific selection of exosomes. A small sample size was initially used to characterize the exosomes and measure AChE activity in CD9 or L1CAM positive exosomes. Based on the preliminary test results, a larger number of participants were included to analyze the differences between the PD and HC groups. Furthermore, we investigated the correlation between exosomal AChE activity and the clinical indicators of PD.

## 2 Method

### 2.1 Participants and sampling

Between March 2020 and June 2022, 44 HC and 37 patients with PD were recruited from the Veterans Health Service Medical Center. PD was diagnosed based on the UK Parkinson’s Disease Society Brain Bank criteria. Patients with PD underwent screening using FP-CIT PET, and their condition severity was evaluated using the Hoehn and Yahr (H&Y) criteria and the Unified Parkinson’s Disease Rating Scale (UPDRS). The HC group was composed of individuals without neurological disorders or significant medical conditions. All participants underwent the Mini-Mental State Examination (MMSE) to assess cognitive impairment. Prior to their involvement, all participants or their legal representatives provided written informed consent, and the study protocol was approved by the Institutional Review Board of the Veterans Healthcare Medical Center (2019–05-004).

### 2.2 Collection and processing of plasma

Blood samples were collected in tubes containing EDTA, as an anticoagulant. To avoid the contamination of AChE derived from red blood cells, samples exhibiting hemolysis were excluded. Subsequently, the samples were centrifuged at 1500 × g for 10 min to remove the red blood cells, platelets, and cellular debris. The separated plasma was promptly transferred to tubes and kept at −80°C until further use.

### 2.3 Magnetic bead-antibody conjugation

Streptavidin beads were mixed with biotinylated CD9 (13-0098-82, eBioscience) or L1CAM (GTX40148, Genetex) antibodies. The mixture was incubated at room temperature for 30 min to conjugate the antibodies to the beads. Following the incubation, the antibody-conjugated beads were washed four times with phosphate-buffered saline (PBS) containing 0.1% BSA. The beads were then resuspended and stored at 4°C until further use.

### 2.4 Exosome isolation

Exosomes were isolated from plasma using antibody-conjugated magnetic beads. Plasma was subjected to a series of sequential centrifugation steps at 300 × g for 10 min, 2,000 × g for 20 min, and 10,000 × g for 30 min. The pellet was subsequently discarded, and the collected supernatant was diluted with PBS in a 1:1 ratio. Magnetic beads coated with CD9 or L1CAM antibodies were added to the diluted supernatant, and exosome isolation was performed at room temperature for 3 h. The magnetic beads were collected and washed with PBS containing Tween 20. To lyse the exosomes, the bead suspension in PBS was treated with M-PER buffer (#78501, Thermo Scientific) and incubated at 4°C for 30 min. Finally, the supernatant, excluding the beads, was collected, and stored at −80°C for further analysis.

### 2.5 Western blot

Exosomes isolated from plasma were mixed with loading buffer and boiled at 95°C for 5 min. The exosome samples were then loaded onto SDS-PAGE gels with a concentration range of 4–20% to separate proteins based on their sizes. Proteins were transferred from the gel to the PVDF membrane at 100 V for 60 min. Following the transfer, the membrane was blocked with a blocking solution of 10% skim milk in Tris buffered saline containing Tween 20 (TBST) for 1 h. Subsequently, the membrane was incubated overnight with specific antibodies against Alix (Cell Signaling Technology, #2171), GM130 (Cell Signaling Technology, #12480), L1CAM, and NeuN (Cell Signaling Technology, #24307). The next day, the membrane was washed three times for 10 min each with TBST and incubated with secondary antibodies (anti-mouse #31430, Thermo Scientific; anti-rabbit #1706515, Bio-Rad) for 1 h. After three additional washes with TBST, the membrane was treated with an ECL substrate (Thermo Scientific, USA) to initiate a chemical reaction. The resulting signal from each antibody was visualized using a GBOX Chemi XRQ system (Syngene, UK).

### 2.6 Measurement of AChE activity in exosome

Exosomal AChE activities were measured from the CD9- or L1CAM-positive exosomes using an AChE fluorescence activity kit (#EIAACHEF, Invitrogen, USA). Exosomes were diluted four-fold in 1X Assay buffer, and 100 μL of the diluted solution was dispensed into each well of a black 96-well plate provided with the kit. Furthermore, 50 μL of a mixture containing the AChE substrate and detection reagent dissolved in dry DMSO was added to each well. After incubation at room temperature for 20 min, the fluorescence signals were recorded using a FLUOstar omega microplate reader (BMG LABTECH, Germany).

### 2.7 Total protein quantitation in exosome

A CBQCA Protein Quantitation Kit (#C-6667, Thermo Scientific) was used to determine the protein levels in the exosome samples. The exosomes were diluted in 0.1 M sodium borate buffer, and KCN from the kit was added and thoroughly mixed with the sample. Subsequently, the CBQCA reagent was added to ensure proper mixing with the sample, and the plate was covered with aluminum foil to protect it from exposure to light. After incubation at room temperature, fluorescence signals were measured at 1 h intervals using a FLUOstar omega micro reader.

### 2.8 Transmission electron microscope

The exosome samples were treated with 1% formic acid for 20 min. Next, the exosomes were placed onto formvar carbon film-coated 150 mesh copper grids for 1 min. Excess samples were removed using a filter paper. For negative staining, the grids were exposed to 2% uranyl acetate. Excess solution was eliminated using a filter paper, and imaging of the exosomes was performed using an 80 kV Hitachi H7600 transmission electron microscope (TEM).

### 2.9 Nanoparticle tracking analysis

After treatment with 1% formic acid, exosomes were diluted in 3 mL of particle-free PBS. Nanoparticle tracking analysis (NTA) was performed using a ZetaView instrument (Particle Metrix, Germany) to assess the concentration and size distribution of the particles. Videos capturing the light-refracting particles were recorded under specific conditions, including a fixed temperature of 22°C, 11 positions, three cycles, sensitivity of 85, shutter setting of 100, and frame rate of 60 frames per second (fps); three measurements were taken. The particle number and size distribution were analyzed using the ZetaView Analyze 08.05.16 SP3 software.

### 2.10 Statistical analysis

Statistical analyses and visualizations were conducted using commercial software SPSS 24 (SPSS Inc., USA) and GraphPad Prism 8 (GraphPad, USA). To evaluate the statistical differences between the two groups with normal and non-normal distributions, t-tests and Mann–Whitney U tests were used. The chi-square test was used to assess statistical differences in sex and visual FP-CITPET readings. Correlations between the clinical factors and biomarkers were examined using Spearman’s correlation analysis. Linear regression models adjusted for age and sex were used to assess correlations between parameters, with standardized coefficients (β) and *p* values calculated. A *p* value less than 0.05 was considered statistically significant. Receiver operating characteristic (ROC) curve analysis was performed to assess the accuracy of the diagnostic values of the biomarkers, and the area under the curve (AUC) was calculated. Cut-offs were determined based on the point at which sensitivity and specificity were maximized.

## 3 Results

### 3.1 Participant demographics and clinical characteristics

The demographic characteristics and clinical features of the participants are summarized in Table 1. The PD group had a higher proportion of older individuals and males compared to the HC group. Statistical analyses were adjusted to account for these differences by considering age and sex as covariates. As the study was conducted in a veterans’ hospital, both groups had a higher proportion of male participants. Additionally, the PD group included individuals diagnosed with PDD, which led to a lower median MMSE score than that of the HC group. All patients with PD underwent FP-CIT-PET, which confirmed the reduced dopamine transport activity. In the HC group, FP-CIT-PET scans were performed on 17 individuals who demonstrated normal dopamine transport activity.

**Table 1:** Demographic and clinical data summarized by diagnostic group.

	PD	HC	*p*
N of individuals	37	44	–
Age	75.0 [73.0–77.5]	73.5 [71.0–76.0]	0.012^b^
Sex [Male/Female]	35/2	32/12	0.016^c^
MMSE	25.0 [19.5–26.0]	26.5 [26.0–28.0]	<0.0001^b^
H&Y score (1/1.5/2/2.5/3)	10/7/14/3/3	–	–
UPDRS part III	45.6 ± 21.6	–	–
FP-CIT-PET (±)^d^	37/0	0/17	<0.0001^c^

a Data are shown as median [interquartile] or mean ± standard deviations.

b Mann–Whitney U test.

c Chi-square test.

d Limited number of participants who underwent FP-CIT-PET scanning.

### 3.2 Characterization of exosomes isolated from human plasma using magnetic beads

**Figure 1:**
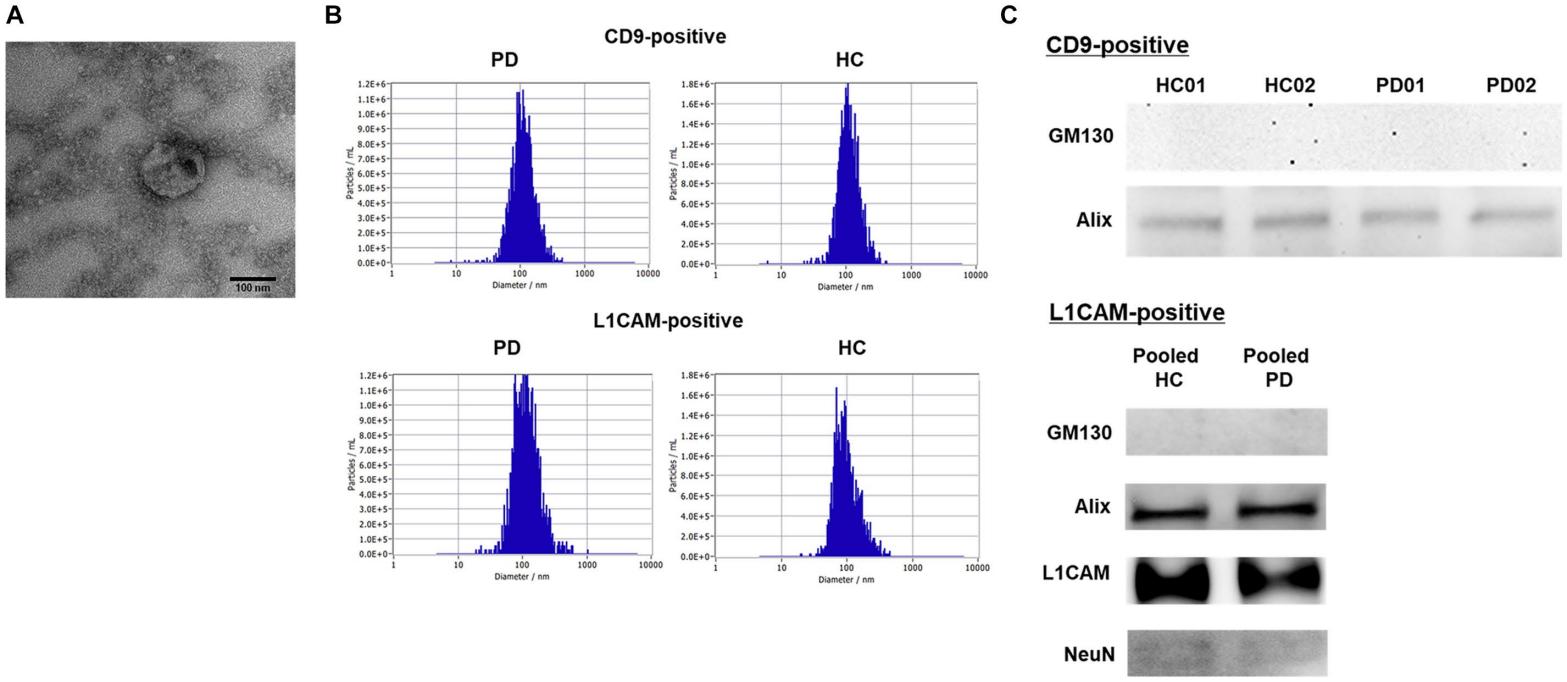
Characteristics of the isolated CD9- or L1CAM-positive exosomes by magnetic beads conjugated with antibodies. **(A)** A representative TEM image of isolated plasma exosomes (Scale bar = 100 nm). **(B)** The distribution of size and concentration of exosomes. **(C)** Western blot in the exosomes derived from PD and HC samples.

Plasma exosomes were isolated using magnetic beads conjugated with antibodies and characterized by NTA, TEM, and western blotting. For NTA, four samples per group were pooled, owing to the limit of detection. TEM imaging revealed the representative morphology of the exosomes (Figure 1A). As shown in Figure 1B, exosomes exhibited a median size of approximately 100 nm (CD9-positive, median size: 106.8 ± 48.1 nm; L1CAM-positive, median size: 110.0 ± 75.0 nm) and the HC (CD9-positive, median size: 107.6 ± 53.4 nm; L1CAM-positive, median size: 91.4 ± 57.7 nm). The concentrations of exosomes were higher in CD9-positive exosomes (PD, concentration: 1.2 × 10^9^ particles/mL; HC, concentration: 0.83 × 10^9^ particles/mL) than in L1CAM-positive exosomes (PD, concentration: 1.8 × 10^8^ particles/mL; HC, concentration: 1.8 × 10^8^ particles/mL). To confirm the successful isolation and purification of exosomes using the magnetic beads, western blot analysis was conducted using specific markers, including Alix (an exosome marker) and GM130 (a negative marker). The results showed a positive signal for Alix, indicating the presence of exosomes, whereas no signal was detected for GM130 (Figure 1C). On the other hand, L1CAM-positive exosomes were pooled from each group owing to low sensitivity. L1CAM displayed a strong signal, and the neural marker NeuN showed a signal, though it was weak (Figure 1C). These findings confirm the successful isolation and purification of exosomes from human plasma using magnetic beads, ensuring the absence of contamination from other plasma proteins.

### 3.3 AChE activities in CD9- and L1CAM-positive exosomes

**Figure 2:**
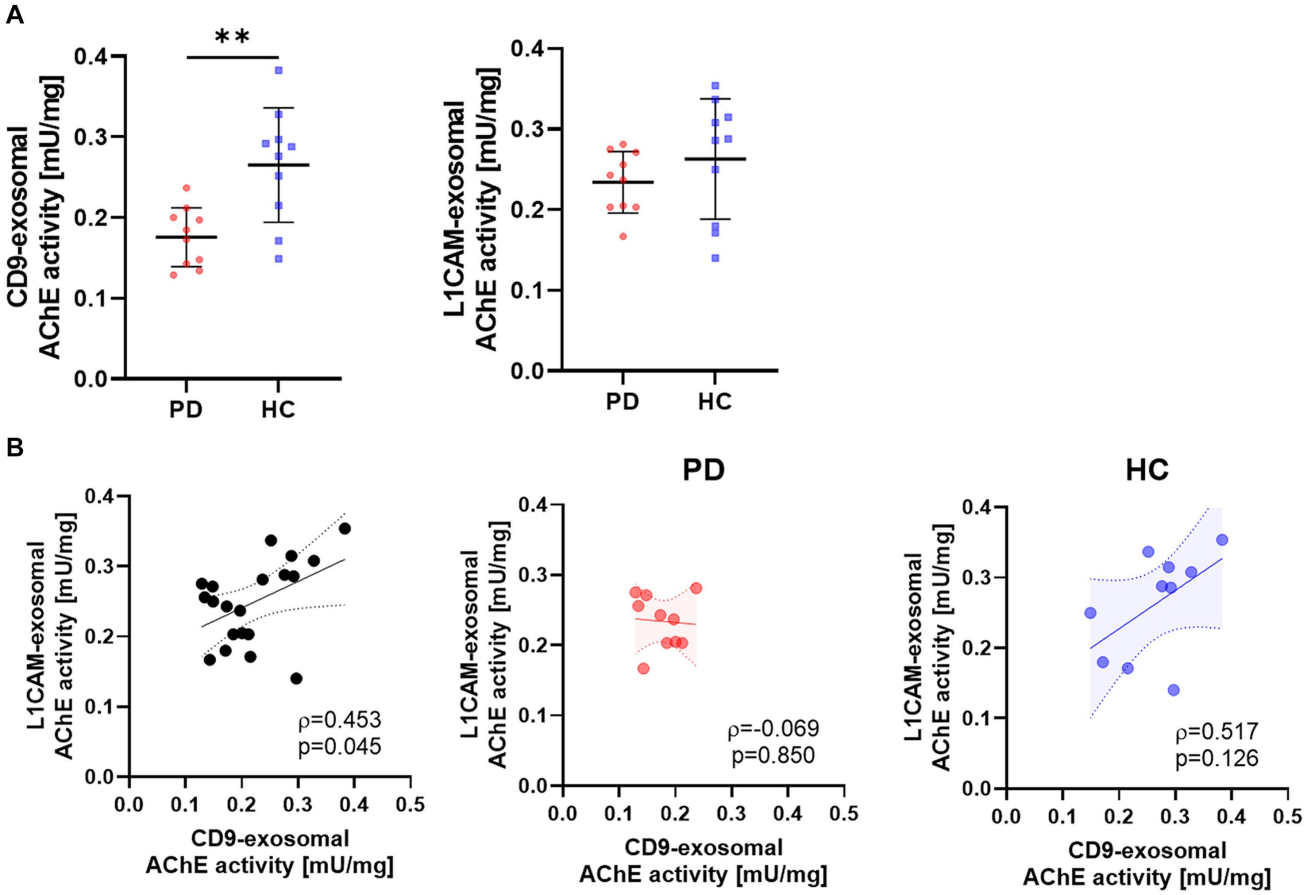
The AChE activities of CD9- and L1CAM-positive exosomes in the PD and HC groups. **(A)** Comparison of AChE activities in CD9- and L1CAM-positive exosomes derived from the PD (n = 10) and HC (n = 10) groups. The horizontal lines are the mean, and vertical lines with short horizontal lines represent the standard deviations. **(B)** Correlation analysis of AChE activities in CD9- and L1CAM-positive exosomes. The dashed lines represent the 95% prediction interval of the regression line. ρ, Spearman’s rho. Spearman’s correlation analysis was used to determine statistical significance. **p < 0.01.

In a preliminary test, the exosomal AChE activity was measured using a limited number of samples to determine the differences between CD9- and L1CAM-positive exosomes in the PD and HC groups. Considering inter-individual differences in exosome quantities, AChE activity was normalized to exosomal protein levels. The results showed that the CD9-positive exosomal AChE activity was lower in the PD group than in the HC group (Figure 2A). Although there was a trend toward reduced AChE activity in the L1CAM-positive exosomes derived from the PD group, no significant differences were observed. Additionally, the AChE activity of CD9- and L1CAM-positive exosomes exhibited a weak positive correlation (Figure 2B). When divided into two groups, this correlation remained a trend in the HC group but was disrupted in the PD group.

**Figure 3:**
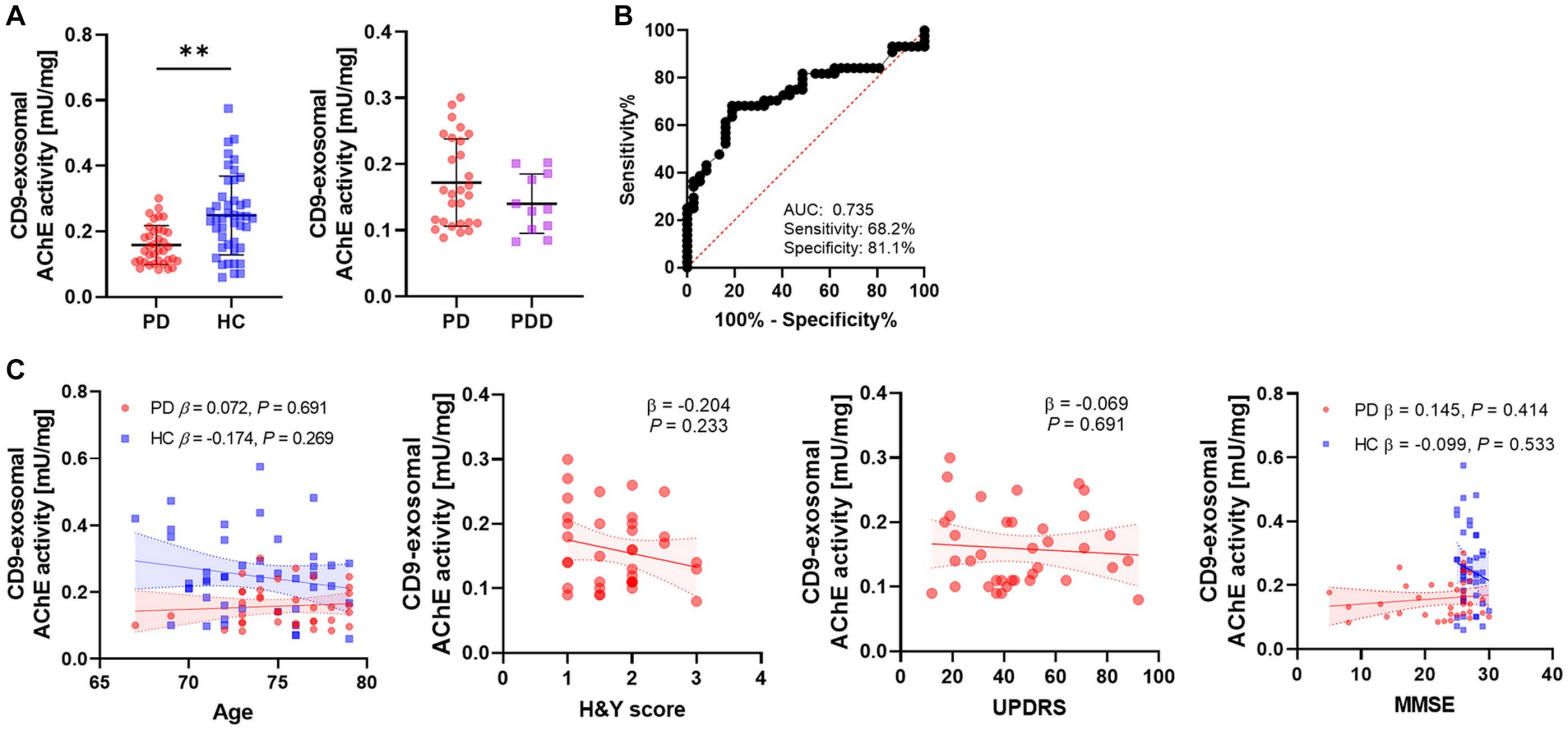
Differences and diagnostic accuracy of the AChE activities in different groups and their correlation with clinical data. **(A)** The AChE activity of CD9-positive exosomes derived from the PD and HC groups. The PD group was classified as PD and PDD according to the presence of the cognitive decline. The horizontal lines indicate the mean, and vertical lines with short horizontal lines are the standard deviations. *P* values derived from binary logistic regression adjusted for age and sex. **(B)** ROC analysis of AChE activity between the PD and HC groups. **(C)** Correlation of AChE activity with age, H&Y scores, UPDRS, and MMSE. *P* values derived from linear regression adjusted for age and sex. Linear regression lines with 95% confidence interval (CI) (dashed lines) are derived from unadjusted models. **p < 0.01.

The AChE activity was measured in CD9-positive exosomes derived from a large number of individuals. The AChE activity in the PD group was significantly lower than that in the HC group (*p* = 0.002; PD, 0.159 ± 0.059 mU/mg; HC, 0.249 ± 0.121 mU/mg; Figure 3A). When the PD group was divided into two subgroups based on the presence of cognitive impairment, there was no difference observed in the AChE activity (PD, 0.169 ± 0.063 mU/mg; PDD, 0.136 ± 0.046 mU/mg). The diagnostic accuracy of AChE activity was 0.742 for AUC, with 68.2% of sensitivity and 81.1% of specificity (Figure 3B). There was no correlation between AChE activity and clinical parameters, including age, H&Y score, UPDRS score, or MMSE score (Figure 3C). Although a negative correlation trend was observed between H&Y score and the AChE activity, it had no statistical significance (β = −0.204, *p* = 0.233).

## 4 Discussion

Exosomes have demonstrated significant utility in liquid biopsy because of their widespread presence in various body fluids and inherent stability conferred by lipid bilayer encapsulation (Zhou et al., 2020). These advantages have led to extensive research into harnessing exosomal markers to understand diseases (Younas et al., 2022). In our previous study, we demonstrated a reduction in AChE activity in plasma-derived exosomes isolated via ultracentrifugation, indicating the potential of exosomes to provide insights into cholinergic dysfunction in PD (Shim et al., 2021). Given the potential for protein aggregation and the difficulties in isolating organ-specific exosomes using ultracentrifugation, we used antibody-coupled magnetic beads for exosome isolation from plasma. Intriguingly, a preliminary study indicated that L1CAM-positive exosomes showed no difference in AChE activity between the PD and HC groups. In contrast, CD9-positive exosomes from the plasma of patients with PD exhibited a substantial reduction in AChE activity with moderate diagnostic accuracy. AChE activity in CD9-positive exosomes was not associated with cognitive impairment but tended to correlate with PD progression.

CD9 is a tetraspanin protein enriched in the exosomal membrane and is considered a canonical marker for exosomes (Jeppesen et al., 2019). In contrast, L1CAM has been widely used for the specific isolation of exosomes derived from the central nervous system (CNS) in blood samples. In this study, AChE activity was measured in L1CAM-positive exosomes based on a previous finding indicating altered levels of α-synuclein in exosomes from patients with PD. However, no significant difference in AChE activity was observed between the PD and HC groups in L1CAM-positive exosomes. Interestingly, a significant reduction in AChE activity was observed specifically in CD9-positive exosomes derived from patients with PD. These findings may be related to the decreased ^11^C-donepezil PET signal observed in peripheral organs, such as the small intestine, colon, and kidney, in patients with PD (Tatyana et al., 2017). Although PD is primarily characterized as a dopaminergic disorder, cholinergic denervation can also occur in PD and is frequently more pronounced than in Alzheimer’s disease (Bohnen et al., 2003; Müller and Bohnen, 2013). The progressive decline in dopamine levels contributes to the impairment of striatal cholinergic interneuron activity (McKinley et al., 2019). Clinical manifestations of PD, such as motor symptoms and gait dysfunction, have also been associated with cholinergic dysfunction (Perez-Lloret and Barrantes, 2016). Consistent with previous studies, our findings demonstrated a significant decrease in AChE activity in the PD group. Given the decreased AChE activity in CD9-positive exosomes, as opposed to L1CAM-positive exosomes, the reduction in AChE activity observed in the blood exosomes of patients with PD may reflect dysfunction within the peripheral rather than the central system.

The underlying mechanism behind the decrease in AChE activity in CD9-positive exosomes and its relationship with PD pathology remain unclear. One possible explanation is that it is a consequence of cholinergic dysfunction in peripheral organs such as the liver, heart, lungs, and gastrointestinal tract, which release CD9-positive exosomes. Salivary glands, which are composed of various cell types, including epithelial cells, can release CD9-positive exosomes (Zlotogorski-Hurvitz et al., 2016). Impaired cholinergic function in the salivary glands may contribute to the development of xerostomia, a common oral manifestation of PD (Cersosimo et al., 2011). The gastrointestinal tract is a potential source of CD9-positive exosomes (Barteneva et al., 2017). Cholinergic dysfunction within the gastrointestinal tract has been implicated in explaining non-motor symptoms, including gastrointestinal dysmotility and constipation, in PD (Tatyana et al., 2017). Similarly, urinary exosomes also exhibit strong CD9 expression (Man Zhang et al., 2021), and the impairment of cholinergic function in the urinary system may be associated with urinary symptoms such as urgency, retention, and incontinence in PD (Sheyn et al., 2022). Finally, although the presence of CD9 in exosomes released by cardiovascular cells has not been established, cholinergic dysfunction in the cardiovascular system can lead to autonomic dysfunction, including orthostatic hypotension, characterized by a drop in blood pressure (Palma and Kaufmann, 2020). These peripheral organs, along with their potential involvement in CD9-positive exosome secretion, provide intriguing perspectives for further investigations to better understand the association between cholinergic dysfunction and PD pathology.

Interestingly, the expression of CD9 is the highest in early erythrocytes and gradually decreases as erythrocytes mature (Isern et al., 2010). During the development of erythrocytes in the bone marrow, exosomes are released (Kuo et al., 2017), which may carry CD9 on their surface along with other proteins and molecules. Previous studies have reported the close relationship between erythrocytes and PD, particularly in relation to α-synuclein. Erythrocytes serve as a major source of α-synuclein in the bloodstream, with approximately 98% of α-synuclein in whole blood being contained within erythrocytes (Barbour et al., 2008; Shi et al., 2010). Notably, under inflammatory conditions, α-synuclein-rich exosomes released by erythrocytes have been shown to cross the blood–brain barrier and contribute to microglial activation and inflammation (Matsumoto et al., 2017). Moreover, increased levels of oligomeric α-synuclein were observed in the erythrocytes of patients with PD compared to HC (Daniele et al., 2018). Another study reported a significant increase in phosphorylated α-synuclein in the erythrocytes of patients with PD and suggested phosphorylated α-synuclein originating from the brain could be secreted into the plasma and taken up by erythrocytes (Matsumoto et al., 2017; Li et al., 2021). These findings imply that erythrocytes and other peripheral organs play a crucial role in the decreased AChE activity observed in CD9-positive exosomes in patients with PD. Further studies are required to investigate the association between α-synuclein and AChE activity in erythrocytes during PD pathogenesis. Additionally, it is important to confirm the origin of the CD9-positive exosomes isolated in this study to determine whether they were derived from erythrocytes.

This study had several limitations. First, the cohort from the veterans’ hospital was biased toward male participants, which may introduce sex-related confounding effects. To minimize the impact of sex, we adjusted for sex as a covariate in all analyses. Second, this was a preliminary study conducted with a limited sample size. Further validation using a larger cohort is necessary to confirm these findings.

## 5 Conclusion

In conclusion, our study demonstrated the potential role of CD9-positive exosomes in PD and cholinergic dysfunction. A significant decrease in AChE activity, specifically in CD9-positive exosomes derived from patients with PD, supports the involvement of peripheral cholinergic dysfunction in this disease. Dysfunction of peripheral organs that exhibit cholinergic functions may contribute to the reduced AChE activity observed in CD9-positive exosomes. These findings suggest that CD9-positive exosomes may provide insights into the underlying pathophysiology. Understanding the relationship among CD9-positive exosomes, cholinergic dysfunction, and PD may have significant implications for diagnostic and therapeutic approaches.

## Data availability statement

The raw data supporting the conclusions of this article will be made available by the authors, without undue reservation.

## Ethics statement

The studies involving humans were approved by Veterans Healthcare Medical Center. The studies were conducted in accordance with the local legislation and institutional requirements. The participants provided their written informed consent to participate in this study.

## Author contributions

SJ: Investigation, Writing – original draft. KS: Conceptualization, Data curation, Methodology, Writing – original draft. DK: Investigation, Writing – review & editing. HB: Investigation, Resources, Writing – review & editing. D-EJ: Investigation, Resources, Writing – review & editing. MK: Funding acquisition, Project administration, Resources, Supervision, Writing – review & editing. SA: Funding acquisition, Project administration, Supervision, Writing – review & editing.
